# NAFLD and MAFLD as emerging causes of HCC: A populational study

**DOI:** 10.1016/j.jhepr.2021.100231

**Published:** 2021-01-19

**Authors:** Shuna Myers, Isabelle Neyroud-Caspar, Laurent Spahr, Konstantinos Gkouvatsos, Evelyne Fournier, Emiliano Giostra, Giulia Magini, Jean-Louis Frossard, Marie-Eve Bascaron, Nathalie Vernaz, Lucia Zampaglione, Francesco Negro, Nicolas Goossens

**Affiliations:** 1 Medical Faculty, University of Geneva, Geneva, Switzerland; 2 Geneva Cancer Registry, University of Geneva, Geneva, Switzerland; 3 Gastroenterology and Hepatology, Geneva University Hospital, Geneva, Switzerland; 4 Division of Transplantation, Geneva University Hospital, Geneva, Switzerland; 5 Division of Palliative Medicine, Department of Rehabilitation and Geriatrics, Geneva University Hospitals, University of Geneva, Geneva, Switzerland; 6 Medical Directorate, Geneva University Hospital, Geneva, Switzerland; 7 Clinical Pathology, Geneva University Hospital, Geneva, Switzerland; 8 Department of Medicine, University of Geneva, Geneva, Switzerland

**Keywords:** Liver cancer, Hepatocellular carcinoma, Metabolic syndrome, Fatty liver, Women’s health, AFP, alpha-foetoprotein, ALD, alcohol-related liver disease, ALT, alanine transaminase, AST, aspartate aminotransferase, ASI, age-standardised incidence, GGT, gamma-glutamyltransferase, HCC, hepatocellular carcinoma, HR, hazard ratio, INR, international normalised ratio, MAFLD, metabolic-associated fatty liver disease, MELD, model for end-stage liver disease, NAFLD, non-alcoholic fatty liver disease, SIR, standardised incidence ratio, TACE, transarterial chemoembolisation

## Abstract

**Background & Aims:**

There are conflicting data regarding the epidemiology of hepatocellular carcinoma (HCC) arising in the context of non-alcoholic and metabolic-associated fatty liver disease (NAFLD and MAFLD). We aimed to examine the changing contribution of NAFLD and MAFLD, stratified by sex, in a well-defined geographical area and highly characterised HCC population between 1990 and 2014.

**Methods:**

We identified all patients with HCC resident in the canton of Geneva, Switzerland, diagnosed between 1990 and 2014 from the prospective Geneva Cancer Registry and assessed aetiology-specific age-standardised incidence. NAFLD-HCC was diagnosed when other causes of liver disease were excluded in cases with type 2 diabetes, metabolic syndrome, or obesity. Criteria for MAFLD included one or more of the following criteria: overweight/obesity, presence of type 2 diabetes mellitus, or evidence of metabolic dysregulation.

**Results:**

A total of 76/920 (8.3%) of patients were diagnosed with NAFLD-HCC in the canton of Geneva between 1990 and 2014. Between the time periods 1990–1994 and 2010–2014, there was a significant increase in HCC incidence in women (standardised incidence ratio [SIR] 1.83, 95% CI 1.08–3.13, *p* = 0.026) but not in men (SIR 1.10, 95% CI 0.85–1.43, *p* = 0.468). In the same timeframe, the proportion of NAFLD-HCC increased more in women (0–29%, *p* = 0.037) than in men (2–12%, *p* = 0.010) while the proportion of MAFLD increased from 21% to 68% in both sexes and from 7% to 67% in women (*p* <0.001). From 2000–2004 to 2010–2014, the SIR of NAFLD-HCC increased to 1.92 (95% CI 0.77–5.08) for men and 12.7 (95% CI 1.63–545) in women, whereas it decreased or remained stable for other major aetiologies of HCC.

**Conclusions:**

In a populational cohort spanning 25 years, the burden of NAFLD and MAFLD associated HCCs increased significantly, driving an increase in HCC incidence, particularly in women.

**Lay summary:**

Hepatocellular carcinoma (HCC) is the most common type of liver cancer, increasingly arising in patients with liver disease caused by metabolic syndrome, termed non-alcoholic fatty liver disease (NAFLD) or metabolic-associated fatty liver disease (MAFLD). We assessed all patients with HCC between 1990 and 2014 in the canton of Geneva (western Switzerland) and found an increase in all HCC cases in this timeframe, particularly in women. In addition, we found that HCC caused by NAFLD or MAFLD significantly increased over the years, particularly in women, possibly driving the increase in overall HCC cases.

## Highlights

• The burden of HCC arising in the context of non-alcoholic and metabolic-associated fatty liver disease (NAFLD and MAFLD) remains unclear.
• We assessed all HCC cases between 1990 and 2014 in an area of western Switzerland.
• We found a significant increase in overall HCC incidence in women but not in men.
• The proportion of NAFLD- and MAFLD-HCC increased in both sexes, particularly in women.
• Liver function of MAFLD patients was intermediate between ‘pure’ NAFLD and non-MAFLD individuals.

## Graphical abstract

**Figure:**
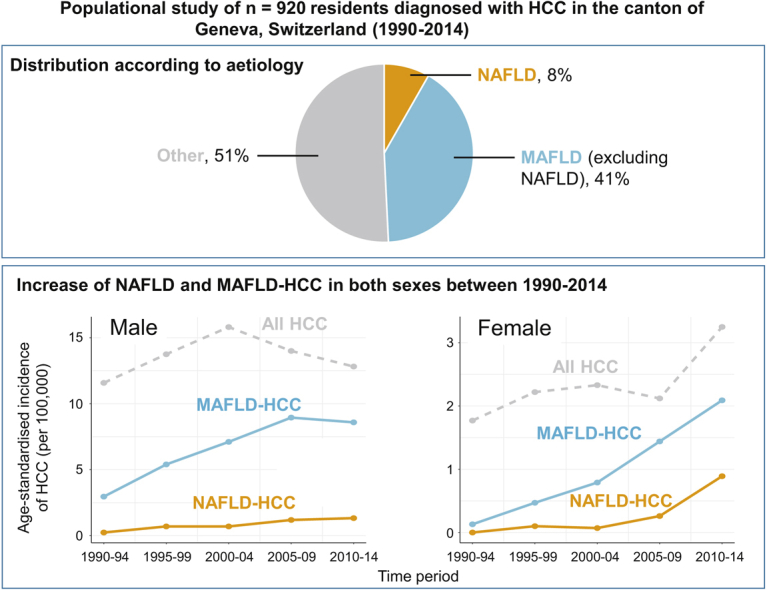

## Introduction

Hepatocellular carcinoma (HCC) is a major global public health challenge ranking sixth in global cancer incidence and fourth in cancer mortality (841,000 new cases and 781,000 deaths worldwide in 2018).^1^ Primary liver cancer, primarily composed of HCC, has a very poor prognosis with a 5-year survival of 17% just behind pancreatic cancer.^2^ Globally, incident cases of liver cancer increased 114% from 471,000 in 1990 to 1,007,800 in 2016 with a most pronounced increase in countries with a high sociodemographic index such as Western Europe, North America, and Australasia.^3^ There exists, however, a significant sex-based disparity for the development of HCC as it arises in men with a male-to-female ratio of approximately 2:4 depending on age, ethnicity, aetiology of liver disease, and other factors.^4^ Nevertheless, HCC incidence and mortality are increasing in both sexes globally. Data from the US Surveillance, Epidemiology, and End Results Program (SEER) highlighted that compared with all primary cancers, from 2008 to 2017, liver cancer had the second highest annual percentage increase in men and the highest annual percentage increase in incidence in women (1.1% and 2.1% annual change in men and women, respectively).^2^ However, drivers of this increase, especially stratified by sex, remain incompletely understood.

Over the past decades, non-alcoholic fatty liver disease (NAFLD) has emerged as the most prevalent chronic liver disease worldwide, in line with the increase of prevalence of features of metabolic syndrome.^5^ NAFLD is generally defined as the presence of hepatic steatosis in >5% of hepatocytes, in the absence of a secondary cause, including significant alcohol consumption.^6^ NAFLD covers a spectrum ranging from simple steatosis, to non-alcoholic steatohepatitis (NASH), liver fibrosis, cirrhosis, and HCC. In view of a better understanding of the pathophysiology of the disease and to develop positive criteria for its diagnosis, a group of experts recently proposed the term metabolic or metabolic dysfunction-associated fatty liver disease (MAFLD) and also suggested an accompanying definition.^7^ The MAFLD concept is, in contrast to NAFLD, not exclusionary and refers to the presence of liver steatosis in addition to overweight/obesity, presence of type 2 diabetes mellitus, or evidence of metabolic dysregulation. In the USA, NASH is the second most common cause of liver transplantation (LT) for HCC and the proportion of NASH in candidates for LT for HCC increased 7.7-fold between 2002 and 2016.^8^ Besides, NASH is the leading cause of LT in women in the USA.^9^ Outside of a LT setting, data from the US SEER registries linked to Medicare showed a 9% increase of NAFLD-HCC from 2004 to 2009^10^ and in Japan the proportion of non-viral aetiologies of HCC (also including alcohol-related liver disease [ALD]) in 53 participating hospitals increased from 10% to 24% between 1991 and 2010.^11^ Nevertheless, many of these studies rely on samples of convenience, are based on tertiary centre referrals, and there is a lack of populational data rigorously assessing the incidence over time of NAFLD-HCC with minimal referral or recall bias and extensive clinical characterisation. In addition, the contribution of NAFLD and especially MAFLD to the HCC burden according to sex remains unclear. We therefore aimed to examine the changing contribution of NAFLD and MAFLD on HCC in a populational study of a well-defined geographical area and highly characterised HCC population between 1990 and 2014.

## Patients and methods

### Study design and setting

We conducted a population-based study between 1990 and 2014, identifying all patients with HCC and analysing temporal trends of incidence and mortality by aetiology of HCC in the resident population of the canton of Geneva, Switzerland (total population in 1990 and 2014 of 382,543 and 482,545, respectively). The canton of Geneva is served by 1 public tertiary referral hospital of 1,890 beds, serving all patients from the canton of Geneva and equipped with an integrated electronic health record.

### Study population

All patients with a diagnosis of HCC between 1 January 1990 and 31 December 2014 were eligible for the study. Case identification was prospectively performed by the Geneva Cancer Registry (GCR), which covers 100% of the population of the canton of Geneva and prospectively collects data on all new arising cancer cases. The GCR was established in 1970, is one of the oldest cancer registries in Europe, and is one of the few registries in the world that collects an extended set of clinical data on diagnosis, stage, treatment, and survival. The GCR has high accuracy, with a very low proportion (<2%) of cancers documented from death certificates.^12^

Diagnosis of HCC was based on standard criteria according to the latest international guidelines at the time of patient inclusion and coded with the diagnostic code C22.0 according to the International Classification of Diseases for Oncology, third edition (ICD-O-3).^13^ Two authors reviewed all diagnoses of HCC and 6 patients who had been misclassified were excluded (Fig. S1).

### Variables

NAFLD was defined by the exclusion of other liver diseases (in particular HCV, HBV, and ALD) and either (1) histological documentation of NAFLD, (2) physician documentation of NAFLD at the time of diagnosis, or (3) the presence of type 2 diabetes, obesity, or metabolic syndrome as defined by the US Expert Panel on Detection, Evaluation, and Treatment of High Blood Cholesterol in Adults.^14^ Ninety-seven percent of NAFLD patients had documented steatosis (histological 52%, biomarker 38%, and radiological 7%). Two patients (2.6%) with NAFLD-cirrhosis had limited clinical data available therefore insufficient data at the time or before diagnosis to document steatosis. We performed a sensitivity analysis of our definition of NAFLD-HCC by including patients with cryptogenic cirrhosis (all major aetiologies of liver disease were excluded; *definition 2*).^8^ The diagnosis of other liver diseases was retrieved from electronic health records and made according to standard diagnostic criteria. Other liver diseases included hepatitis B, hepatitis C, ALD, other rarer causes (including autoimmune liver disease, haemochromatosis, alpha-1 antitrypsin deficiency, and others), no identifiable cause (no criteria for any liver disease, despite adequate work-up), and unknown (no clear aetiology but no complete work-up available). Overall, we could identify the aetiology of liver disease in 88% of men and 83% of women and could assess alcohol consumption in 85% of the study population.

Criteria for MAFLD included one or more of the following 3 criteria: overweight/obesity, presence of type 2 diabetes mellitus, or evidence of metabolic dysregulation.^7^ Ninety-four percent of patients with MAFLD had documented steatosis (histological 53%, biomarker 39%, radiological 7%). Twenty-five patients (5.6%) with MAFLD-cirrhosis had limited clinical data available therefore insufficient data at the time or before diagnosis to document steatosis. Unlike NAFLD, patients with other underlying liver disease aetiologies were also considered to have concomitant MAFLD if they met the criteria.

Liver disease was staged according to fibrosis stage based on either (1) non-tumoural liver biopsy results within 1 year of HCC diagnosis, (2) non-invasive measurements of advanced fibrosis with the FIB-4 score (FIB-4 score over 3.25 suggesting advanced liver disease), or (3) manifest clinical or radiological signs of advanced liver disease such as ascites or gastroesophageal varices.

Additional clinical, biochemical, and radiological data, as listed in Table 1, were collected if present within 1 month of HCC diagnosis. Surveillance for HCC was defined as liver imaging by computed tomography or ultrasound performed to detect HCC in the past 12 months before HCC diagnosis. HCC was prospectively staged by the GCR using the TNM classification. Therapy for HCC was prospectively collected by the GCR and confirmed using patient records. HCC therapy was classified as curative (radiofrequency ablation, percutaneous ethanol injection, resection, LT), palliative (transcatheter arterial chemoembolisation [TACE], selective internal radiation therapy, external beam radiotherapy, systemic therapy by sorafenib) or best supportive care (comprising either no oncologic therapy or systemic therapy other than sorafenib).

**Table 1:** Baseline characteristics of the study population.

Variable	NAFLD, n = 76	Non-NAFLD, n = 844	*p* value
Clinical			
Age (years)	75 (70–80)	67 (59–75)	<0.001
Female sex (%)	24/76 (31.6%)	157/844 (18.6%)	<0.001
Diabetes (%)	54/74 (73%)	237/598 (39.6%)	<0.001
Hypercholesterolaemia (%)	34/72 (47.2%)	100/551 (18.1%)	<0.001
Weight (kg)	81 (69.8–91)	74.7 (64.4–85)	0.005
BMI (kg/m^2^)	28.7 (25.6–31)	25.6 (22.4–28.7)	<0.001
BMI ≥25 kg/m^2^ (%)	48/73 (65.8%)	195/591 (33%)	<0.001
Biological			
ALT (IU/L)	36 (25–61)	49 (31–84)	0.004
AST (IU/L)	53 (37.5–95.2)	83 (53–141)	<0.001
Albumin (g/L)	30 (27–34)	28 (24–33)	0.076
Total bilirubin (μmol/L)	19 (14–31)	54.7 (–24–133.4)	<0.001
Creatinine (μmol/L)	87.5 (73–117.8)	82 (69–105)	0.162
Sodium (mmol/L)	137 (134.8–139)	137 (134–139)	0.225
Platelets (10^9^/L)	224.5 (142.2–305)	138 (89.5–202.5)	<0.001
GGT (IU/L)	167 (62–295)	189.5 (108–360.8)	0.118
Alk Phos (IU/L)	130.5 (82.8–196.8)	129.5 (94–190)	0.739
Haemoglobin (g/L)	126 (107–145.5)	127.5 (110–142)	0.746
INR	1.19 (1.03–1.64)	1.35 (1.12–1.72)	0.010
MELD	10 (8–14)	12 (8–16)	0.044
Background liver			
Histological or clinical F3–F4	41/67 (61.2%)	558/598 (93.3%)	<0.001

Differences between groups were tested using the Mann-Whitney *U* test or Chi-square test as appropriate. ALT, alanine transaminase; AST, aspartate aminotransferase; GGT, gamma-glutamyltransferase; INR, international normalised ratio; MELD, model for end-stage liver disease; NAFLD, non-alcoholic fatty liver disease.

Vital status was recorded at the end of the study period (11 October 2018) by linkage of the electronic chart system to the Cantonal Population Office.^15^

### Statistical analysis

Continuous data are presented as medians with IQRs. Categorical and dichotomous data are expressed as total numbers and percentages. Differences between groups were tested using the Mann-Whitney *U* test or the Chi-square test as appropriate. BMI ≥25 kg/m^2^ was grouped as overweight or obesity. A 2-sided *p* value of <0.05 was considered statistically significant. Estimated resident population data by sex, age group, and year were obtained from the Cantonal Population Office for incidence rate calculations and incidence rates were calculated for the entire cohort and stratified by sex, period, and aetiology subgroups. Age-standardised incidence (ASI) was calculated directly according to the European Standardised population.^12^ All statistics were performed using the R software (version 3.4.4, R core team, R Foundation for Statistical Computing, Vienna, Austria). A *p* value of <0.05 was considered statistically significant.

### Ethical considerations

This study was approved by the ethics committee in Geneva, with a waiver of informed consent owing to the minimal risk to participants, and the disproportionate difficulty in obtaining informed consent linked to the high mortality of HCC. In addition, the prospective collection of data by the GCR is covered by a general authorisation to collect nominative data and analyse anonymised data granted in 1991 by the Federal Expert Commission for data protection and research.

## Results

### Study population

From the total resident population of the canton of Geneva, we identified 926 cases who developed liver cancer from 1990 to 2014. A total of 920 patients with HCC were included after the exclusion of 6 patients: unclear HCC diagnosis (n = 3), hepatocholangiocarcinoma (n = 2) and absence of documented HCC (n = 1; Fig. S1). When assessing the aetiology of liver disease, 76/920 patients (8.3%) had NAFLD-HCC, 397/920 (43.1%) had ALD-HCC, 191/920 (20.7%) had HCV-HCC, 84/920 (9.1%) had HBV-HCC, and 172/920 (18.7%) had HCC from other or unknown causes. As expected, when comparing the 76 patients with NAFLD-HCC with the 844 patients with non-NAFLD-HCC, we found that features of metabolic syndrome such as the presence of type 2 diabetes, obesity, and hypercholesterolemia were more common in patients with NAFLD-HCC (Table 1). Besides, patients with NAFLD-HCC were older, more often female, and had better liver function as evidenced by higher albumin levels, lower international normalised ratio (INR) and model for end-stage liver disease (MELD) scores, whereas higher platelet counts suggested less severe portal hypertension (Table 1).

We next assessed tumour-related characteristics in patients with NAFLD-HCC and non-NAFLD-HCC (Table 2). When compared with non-NAFLD-HCC patients, those with NAFLD-HCC had lower alpha-foetoprotein (AFP), lower classification for the primary tumour (T) and less common metastatic (M) disease leading to a less advanced stage of HCC (Table 2). Interestingly, HCC surveillance in the year before diagnosis was significantly less common in NAFLD-HCC patients (9% *vs.* 37% in non-NAFLD-HCC, *p* <0.001). When assessing therapy for HCC, patients with NAFLD-HCC had a higher proportion of undergoing liver resection than those with non-NAFLD-HCC (21% *vs.* 9%, *p* = 0.007; Table 2).

**Table 2:** Baseline characteristics of the HCC-related variables in the study population.

Variable	NAFLD, n = 76	Non-NAFLD, n = 844	*p* value
AFP (ng/ml)	9.5 (4–463)	27 (6–443.5)	0.032
Surveillance before diagnosis	5/56 (9%)	144/391 (37%)	<0.001
Median survival (weeks)	101 (46–106)	38 (33–44)	0.006∗
Cancer staging			
T1	14/70 (20%)	131/714 (18%)	0.002
T2	26/70 (37%)	197/714 (28%)	
T3	26/70 (37%)	218/714 (31%)	
T4	4/70 (6%)	168/714 (24%)	
N0	41/47 (87%)	325/420 (77%)	0.200
N1	6/47 (13%)	94/420 (22%)	
N2	0/47 (0%)	1/420 (0.2%)	
M0	36/50 (72%)	250/495 (51%)	0.004
M1	14/50 (28%)	245/495 (49%)	
TNM stage			0.030
1 (T1N0M0)	10/50 (20%)	71/495 (14%)	
2 (T2N0M0)	13/50 (26%)	90/495 (18%)	
3 (T3–4N0M0)	13/50 (26%)	89/495 (18%)	
4 (N1 or M1)	14/50 (28%)	245/495 (49%)	
Therapy			
Liver transplantation	2/73 (3%)	51/705 (7%)	0.220
Resection	15/73 (21%)	65/705 (9%)	0.007
RFA	10/73 (14%)	68/705 (10%)	0.303
TACE	32/73 (44%)	268/705 (38%)	0.377
Systemic therapy	10/73 (14%)	102/705 (14%)	1.000
Other	15/73 (21%)	77/705 (11%)	0.020
Best supportive care/no therapy	20/73 (27%)	267/705 (38%)	0.097

Differences between groups were tested using the Mann-Whitney *U* test or Chi-square test as appropriate. ∗Log-rank *p* value. AFP, alpha-foetoprotein; HCC, hepatocellular carcinoma; NAFLD, non-alcoholic fatty liver disease; RFA, radiofrequency ablation; TACE, transarterial chemoembolisation.

Owing to the significantly higher proportion of women among patients with NAFLD-HCC (32% *vs.* 19% among NAFLD-HCC *vs.* non-NAFLD-HCC patients, respectively, *p* < 0.001), we compared the clinical characteristics of men and women with NAFLD-HCC. Overall, clinical, biological, and tumour characteristics were similar (Table S1) except, as expected, for lower weight in female patients. The MELD score was significantly lower in women with NAFLD-HCC compared with men (9 *vs.* 11 respectively, *p* = 0.018). However, this difference may have been driven by lower creatinine levels in women (78 *vs.* 91 μmol/L, *p* = 0.052) and might not reflect better liver function in women as previously published.^16^

### Characterisation of patients with MAFLD-HCC

To assess the impact of the recent proposal updating the current definition of NAFLD with MAFLD we characterised patients with MAFLD-HCC in our cohort. Firstly, we compared the clinical characteristics of 3 groups: patients with MAFLD without NAFLD (n = 377), patients with NAFLD (all were also considered to have MAFLD, n = 76), and individuals without NAFLD or MAFLD (n = 467; Table S2). As expected, the features of metabolic syndrome were more prevalent in the NAFLD/MAFLD groups compared with the non-MAFLD group. Interestingly, the bilirubin and platelet counts of the patients with MAFLD without NAFLD were significantly different from both the NAFLD group (total bilirubin: 26 *vs.* 19 μmol/L, *p* = 0.001 and platelets: 144 *vs.* 225×10^9^/L, *p* <0.001, respectively) and the non-MAFLD group (total bilirubin: 26 *vs.* 32 μmol/L, *p* = 0.030 and platelet count 144 *vs.* 129×10^9^/L, *p* = 0.033) suggesting that the severity of underlying liver disease and portal hypertension of those with MAFLD without NAFLD was somewhat in between the 2 other groups. This reflected in a lower proportion of patients with HCC who had best supportive therapy or no therapy in the MAFLD group compared with the group without MAFLD (32% *vs.* 45%, *p* <0.001).

To further characterise these differences, we compared patients with and without MAFLD across HCV and ALD patients, the 2 major aetiologies of HCC in our population (Table S3). The proportion of patients with MAFLD was significantly higher in the ALD group (55% *vs.* 43% for ALD and HCV, respectively, *p* = 0.005). Interestingly, the differences described above were particularly prominent when comparing ALD-MAFLD *vs.* ALD-without MAFLD with lower serum total bilirubin (27 *vs.* 41 μmol/L, *p* = 0.002), higher albumin (29 *vs.* 27 g/L, *p* = 0.003) and lower MELD (12 *vs.* 13, *p* = 0.020) suggesting better liver function in patients with ALD-HCC and MAFLD. This also reflected in significantly fewer patients undergoing best supportive therapy or no therapy (37 *vs.* 54%, *p* = 0.003). These differences were less notable in patients with HCV although the HCV population was smaller and these analyses may have been under-powered.

### Incidence of HCC

**Fig. 1:**
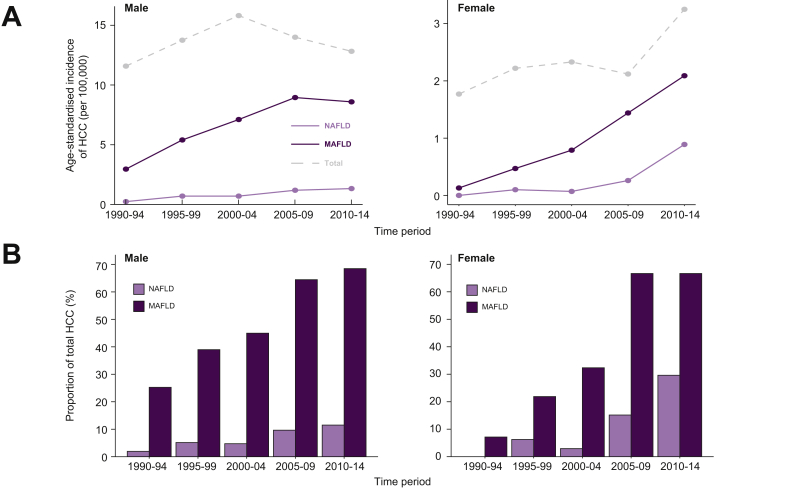
**Burden of NAFLD-HCC and MAFLD-HCC between 1990 and 2014 in the canton of Geneva.** (A) Age-standardised incidence of NAFLD-HCC, MAFLD-HCC and total cases in the canton of Geneva in males and females in 5-year periods from 1990 to 2014. (B) Proportion of NAFLD-HCC and MAFLD-HCC over all HCC by sex between 1990 and 2014. HCC, hepatocellular carcinoma; MAFLD, metabolic-associated fatty liver disease; NAFLD, non-alcoholic fatty liver disease.

We next assessed the progression of ASI of HCC between 1990 and 2014 in 5-year periods (Fig. 1) stratified by sex. In men, ASI of HCC in the canton of Geneva increased non-significantly between the period 1990–1994 to 2010–2014 from 11.6 to 12.8 per 100,000 (standardised incidence ratio [SIR] 1.10, 95% CI 0.85–1.43, *p* = 0.468). In women, ASI of HCC increased significantly from 1.8 to 3.3 per 100,000 (Fig. 1A; SIR 1.83, 95% CI 1.08–3.13, *p* = 0.026).

**Fig. 2:**
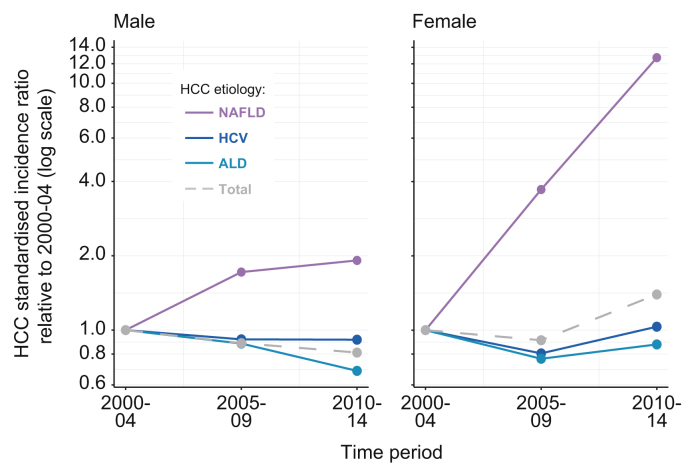
**Standardised incidence ratio for HCC compared with the time period 2000–2004 for NAFLD-HCC, HCV-HCC, ALD-HCC, and total HCC in males and females.** Note the log scale on the y-axis. ALD, alcohol-related liver disease; HCC, hepatocellular carcinoma; NAFLD, non-alcoholic fatty liver disease.

To understand these differences better, we assessed aetiology-specific changes in incidence. In men, NAFLD-HCC ASI increased from 0.24 (95% CI 0.03–0.9) to 1.36 (95% CI 0.8–2.2) per 100,000 with the share of NAFLD-HCC increasing significantly from 2% to 12% between 1990–1994 and 2010–2014 (*p* = 0.010, Fig. 1B). In the same timeframe, in women with NAFLD-HCC, ASI increased from 0.0 (95% CI 0.0–0.39) to 0.89 (95% CI 0.49–1.5) per 100,000 with the proportion of NAFLD-HCC increasing from 0% to 29% (*p* = 0.037, Fig. 1B). NAFLD remained the fourth most common aetiology from 1990–1994 to 2010–2014 in men, whereas in women NAFLD increased in the same period from fourth place to second place, just behind HCV (Fig. S2). We then compared the SIR of NAFLD-HCC compared with the period 2000–2004 for the 3 major aetiologies of HCC in our area (Fig. 2). We chose as a baseline 2000–2004 as our electronic health record was fully implemented at that time leading to a significant reduction in patients with unknown aetiology of HCC after 2000. In men, we found a decrease in incidence for ALD-related HCV-related and all cause HCC ASI from 2000–2004 to 2010–2014 (SIR of 0.81, 0.91, and 0.68 compared with 2000–2004, respectively). However, NAFLD-HCC incidence nearly doubled in the same period in men (SIR 1.92, 95% CI 0.77–5.08). In women, total HCC increased in line with a major increase in NAFLD-HCC incidence (SIR 1.39 [95% CI 0.31–193] and 12.7 [95% CI 1.63–545] in 2005–2009 and 2010–2014, respectively).

In both sexes, between 1990 and 2014 the ASI of HCC associated with MAFLD increased from 1.30 (95% CI 0.75–2.10) to 5.03 (95% CI 4.01–6.23) per 100,000 with a SIR of 3.86 (95% CI 2.26–7.02) with a particularly striking increase in women with a more than 16 times increase in the same timespan (SIR 16.1 [95% CI 2.45–483]; Fig. 1A). Over the same period, the proportion of MAFLD-associated HCCs increased from 21% to 68% (*p* <0.001), with a steeper increase in women from 7% to 67% (*p* <0.001; Fig. 1B). When comparing non-NAFLD aetiologies of HCC, there was a significantly higher proportion of MAFLD in ALD-HCC than HCV-HCC (49% *vs.* 35%, *p* = 0.003).

The increase of NAFLD- and MAFLD-related HCC are in line with an increase of the prevalence of overweight or obesity in the canton of Geneva between 1992 to 2012 in men (42% increase in overweight or obesity and 123% increase in obesity prevalence) and women (92% increase in overweight or obesity and 126% increase in obesity prevalence; Fig. S3).^17^

### Sensitivity analysis

Owing to the lack of unified diagnostic criteria of NAFLD-HCC, we used restrictive criteria for NAFLD-HCC diagnosis (see Patients and methods). To assess whether our findings were robust when the definition was expanded to include patients with cryptogenic liver disease, we performed a sensitivity analysis (definition 2, see Patients and methods), leading to an increase of 25 (33%) of cases of NAFLD-HCC (definition 1, n = 76; definition 2, n = 101). Broadly, characteristics of both definitions of NAFLD-HCC were well matched except, as expected, for a lower proportion of diabetics (Table S4). Similarly to our base definition of NAFLD-HCC, based on definition 2, between the periods 2000–2004 and 2010–2014 there was a significant increase in NAFLD-HCC cases in women (SIR 9.29, [95% CI 1.69–98.2]) but not in men (SIR 1.40 [95% CI 0.65–3.17]; Fig. S4).

## Discussion

Previous studies have attempted to longitudinally assess the burden of NAFLD-HCC. For instance, a study assessing the temporal trends of HCC between 2004 and 2009 in the USA showed an 11% annual increase of all HCC cases and a 9% annual increase for NAFLD-HCC.^10^ However, the study was not populational as it was based on US SEER registries linked to Medicare and the aetiology of liver disease was based on ICD-9 which lacks a NAFLD code and may thus misclassify the aetiology of underlying liver disease.^18^ A large retrospective cohort in US Veterans Administration patients also found an increase in overall HCC incidence between 2001 and 2013; however, it failed to show a clear increase in NAFLD-HCC incidence but this study had similar limitations to the previous one as it also based NAFLD diagnosis on ICD-9 diagnostic codes and was not populational.^19^ Another monocentric UK-based study found a more than 10-fold increase in NAFLD-HCC referrals to a centralised specialist team with a 1.8-fold increase in HCC mortality between 2000 and 2010 and a high prevalence of metabolic risk factors but this study was also limited by potential referral bias.^20^ A Japanese multicentric study also found that the proportion of patients with non-viral HCC increased from 10% in 1991 to 24% in 2010.^11^ However, again this study was not populational but based on the cohorts of participating centres and when assessing the aetiologies of these non-viral HCCs only 11% were considered to be NAFLD and 54% were unclassified suggesting again a number of undiagnosed NAFLD-HCCs. Overall, these studies suggest that there is an increase of overall HCC incidence in a variety of epidemiological settings throughout the world and that there may be an increase in NAFLD-HCC incidence; however, none of these studies had a populational approach covering a specific population with clear and well-defined criteria for diagnosis of NAFLD. In addition, to our knowledge, no study assessed the burden of MAFLD-HCC. We therefore aimed to examine the changing contribution of NAFLD and MAFLD on HCC in a populational study of a well-defined geographical area and highly characterised HCC population in a timespan of 25 years.

Overall, we found an increase in HCC ASI between 1990 and 2014, with a particularly marked increase in women. This increase is in line with HCC trends in several low-risk countries of Europe, the Americas, Africa, Oceania, and India, possibly linked to an increase in NAFLD-HCC, although other high-risk countries globally have seen a decline in HCC incidence.^21^ There has been an increase in HCC mortality in both sexes between 2002 and 2012 in Northern and Central Europe and the USA but a decrease in Southern Europe in line with control of viral hepatitis and increased overweight/obesity and diabetes.^22^

We found that compared with other aetiologies of HCC, there was a clear increase of the burden of NAFLD-HCC, particularly in women. In line with an increasing prevalence of metabolic syndrome in the canton of Geneva, we found an increase in NAFLD-HCC incidence in men and women over a period of 25 years. Previous reports have highlighted the rising burden of NAFLD on the development of liver disease and liver cancer. For instance, the proportion of NAFLD increased from 2.6% to 19.5% from 1995–1999 to 2010–2014 in a series of surgical resections for HCC in 2 centres in Paris^23^ and there was a more than 10-fold increase in NAFLD-HCC referrals between 2000 and 2010 to a tertiary referral centre in the UK paralleling an increase in regional HCC mortality.^20^ Modelling studies have also suggested that the burden of HCC linked to NAFLD may increase exponentially in the USA and Europe.^24^^,^^25^ However, as previously discussed, previous studies have had a number of limitations potentially not capturing the burden of NAFLD-HCC at a populational level. To our knowledge, our study is the first that captures the burden of overall HCC and NAFLD-HCC, stratified by aetiology, in a well-defined population at intermediate HCC risk over 25 years.

A group of experts have recently proposed that a novel definition of MAFLD better encompasses the burden of metabolic dysfunction on liver disease than previously used definitions and this change in nomenclature has been endorsed by a number of regional associations.[26], [27], [28] However, this change remains controversial and its consequences, notably in the context of complications such as HCC, remain unclear.[29], [30], [31] To assess the potential role of MAFLD diagnosis in the context of HCC we compared characteristics of patients with MAFLD across subgroups. We found that a number of characteristics of patients with MAFLD-HCC (excluding NAFLD) were distinct from NAFLD and non-MAFLD. For instance, liver function and severity of portal hypertension of MAFLD-HCC was intermediate between the 2 other groups as was stage of liver cancer. These factors led to a lower proportion of MAFLD-HCC who only had palliative or no therapy compared with individuals who did not have MAFLD. Interestingly, these differences were especially notable in patients with MAFLD-ALD and not patients with MAFLD-HCV, although these subgroup analyses may have been under-powered. This suggests that patients with MAFLD, in the setting of HCC, comprise a heterogeneous group of individuals but with overall clinical and tumour characteristics on a continuum between patients with ‘pure’ NAFLD and non-MAFLD. In our analysis, we also identified a significant increase in HCC cases associated with MAFLD over the study period, increasing from 21% to 68% from 1990–1994 to 2010–2014, while the prevalence of overweight/obesity in our region increased from 26% to 41%^17^ between 1992 and 2012. This parallel increase reinforces the well-established association between obesity, metabolic syndrome, and HCC development.^32^ For example, in a prospective cohort of more than 900,000 US adults followed up for 16 years, obesity was significantly associated with increased risk of death from liver cancer and in a longitudinal cohort study including 578,700 individuals, obesity and metabolic syndrome predicted the development of HCC over a mean follow-up of 12 years.^33^^,^^34^ To our knowledge, our cohort is the first report longitudinally examining the burden of MAFLD in patients with HCC in a well-specified region and highly characterised population.

Despite a lower absolute incidence of HCC in women, we found that during the 25 years of the study, the increase in HCC incidence was higher in women when compared with men. In addition, we found that the proportion of NAFLD-HCC was higher in women, reaching nearly 30% of all HCCs in 2010–2014 and thus driving an increase in total HCC incidence. There was a similar proportion of missing aetiology data in men and women (12% and 17%, respectively), thus making it unlikely that different rates of NAFLD misdiagnosis explain this finding. Globally, the incidence of liver cancer, predominantly comprised of HCC, is 2–3 times higher among men in most world regions.^1^ Multiple factors have been postulated to explain this difference, including variations in risk factors, sex steroid hormones, adiponectin levels leading to distinct molecular gene and pathways alterations.[35], [36], [37] However, data from the USA, where the obesity prevalence is among the highest worldwide, have shown that the population attributable fraction for metabolic disorders to develop HCC was similar in men and women and type 2 diabetes was associated with an even higher adjusted hazard ratio of developing HCC in women compared with men.^38^^,^^39^ Data from the SEER also showed that liver cancer was associated with the highest annual percentage increase in cancer in women between 2008 and 2017,^2^ whereas LT listings in the USA between 2004 and 2016, increased 97% for NASH, 1413% for HCC and NASH, and reached a 2383% increase for women with HCC and NASH in the same time period.^9^ Similarly, in our cohort we found a relative increase of female HCC burden with a decrease of overall male/female (M/F) ratio over the years, especially for NAFLD-HCC (in 2010–2014 M/F ratio of 1.5, 2.4, and 10.5 for NAFLD, HCV, and ALD, respectively). This may suggest that in line with the increase of population attributable fraction associated with other obesity-related cancers in women, the increase of NAFLD and MAFLD may lead to a future rise in the relative burden of HCC, particularly in women.^40^

Potential limitations inherent to our approach include the specific setting of our study in a well-specified region in Western Europe and the absence of universal criteria for the diagnosis of NAFLD. Although a potential limitation to our approach includes the specific setting of our study in a well-specified region in Western Europe, we believe that our results have external validity and underline global trends in the epidemiology of HCC for the following reasons. Firstly, Switzerland and the Geneva canton are situated in a region at intermediate risk of HCC with a similar distribution of HCC aetiology seen in other western countries at low-to-intermediate HCC risk.^41^^,^^42^ Secondly, despite an overall low prevalence of overweight/obesity and type 2 diabetes (respectively, 41% and 6% in 2017^17^), the increasing trend is similar to most countries part of the Organisation for Economic Co-operation and Development,^43^ Thirdly, our data are in line with a body of evidence underlining the increasing burden of NAFLD and metabolic syndrome on the development of liver cancer, as discussed above. Lastly, large population-based studies have shown similar increases in the prevalence of features of metabolic syndrome in most regions of the world independent of socio-economic factors. For instance, a global pooled analysis of 128.9 million people showed an increase in mean BMI and obesity prevalence in all world regions between 1975 and 2016 with proportional rise being smallest in high-income regions and largest in southern Africa.^44^ A similar study assessing the worldwide epidemiology of diabetes between 1980 to 2014 found an overall increase, or at best stability in every country of the age-standardised diabetes prevalence leading to a near quadrupling of the number of adults with diabetes worldwide^45^ suggesting that all these regions will also be impacted by the rise in cases of NAFLD-HCC and MAFLD-HCC as seen in our study.

Regarding the definition of NAFLD we defined NAFLD-HCC when specifically stated or when other causes of liver disease were excluded in patients with type 2 diabetes, metabolic syndrome, or obesity and overall, 97% of patients with NAFLD had documented steatosis. To ensure the external and internal validity of our definition of NAFLD, we performed a sensitivity analysis with a broader definition of NAFLD that identified 33% more patients and we noted that characteristics were well matched between the 2 NAFLD definitions and similar longitudinal trends were observed with both definitions. In addition, similar trends were observed when examining MAFLD, irrespective of other aetiology of liver disease, and when assessing the prevalence of the features of metabolic syndrome in our region, suggesting that our approach was consistent with the epidemiology of the disease.

Overall, in a well-characterised populational study spanning 25 years, we found an increasing incidence of NAFLD- and MAFLD-HCC, driving an increase in HCC incidence, particularly in women. Strengths of our approach include a populational approach capturing 100% of the population of the canton of Geneva by the GCR, a detailed clinical characterisation of all study participants allowing identification of patients with NAFLD and MAFLD and the duration of the study spanning 25 years. Future research should aim to assess the impact of NAFLD and MAFLD-HCC in other populations and better characterise epidemiologically and biologically the link between metabolic syndrome and the development of HCC, particularly in women.

## Financial support

This investigation was supported by a grant of the 10.13039/501100003360Geneva Cancer League, the FLAGS foundation and 10.13039/501100006388Geneva University Hospital and 10.13039/501100006389University of Geneva fellowship grants.

## Authors’ contributions

Study concept and design: SM, IN, NG. Study supervision: NG. Acquisition of data: SM, IN, KG, MB, LZ, NV. Statistical analysis: EF, NG. Analysis and interpretation of data: LS, EG, JLF, FN, NG. Obtained funding: NG. Critical revision of the manuscript for important intellectual content: all authors. Final approval of the published version: all authors

## Data availability statement

Raw data are unsuitable to post publicly owing to confidentiality and legal requirements.

## Conflict of interest

The authors declare no conflicts of interest that pertain to this work.

Please refer to the accompanying ICMJE disclosure forms for further details.
