# Supplementary material for: NAFLD and MAFLD as emerging causes of HCC: A populational study
Source: JHEP Rep. 2021 Jan 19;3(2):100231. doi: 10.1016/j.jhepr.2021.100231 (PMC7957147; doi:10.1016/j.jhepr.2021.100231)
Supplement: Supplementary information.pdf [file mmc1.pdf]

NAFLD and MAFLD as emerging causes of HCC: A populational study

Shuna Myers, Isabelle Neyroud-Caspar, Laurent Spahr, Konstantinos Gkouvatsos, Evelyne Fournier, Emiliano Giostra, Giulia Magini, Jean-Louis Frossard, Marie-Eve Bascaron, Nathalie Vernaz, Lucia Zampaglione, Francesco Negro, Nicolas Goossens

Table of contents

Table S1 2
Table S2 4
Table S3 5
Table S4 7
Fig. S1 8
Fig. S2 9
Fig. S3 10
Fig. S4 11

Supplementary Tables

Variable	Male sex, n=52	Female sex, n=24	p-value
**Clinical**			
Age (y)	75 (70-79)	75.5 (65.5-80.5)	0.960
Diabetes (%)	37/50 (74%)	17/24 (70.8%)	0.786
Hypercholesterolemia (%)	23/48 (47.9%)	11/24 (45.8%)	1.000
Weight (kg)	86 (75.8-91.2)	70.5 (64-89.2)	0.025
BMI (kg/m^2)	29.8 (26.7-30.8)	28.1 (24.5-32.3)	0.404
BMI ≥ 25kg/m^2 (%)	34/49 (69.4%)	14/24 (58.3%)	0.433
**Biological**			
ALT (IU/L)	36.5 (25-56.8)	35 (24.8-69.8)	0.976
AST (IU/L)	53 (38.8-82.8)	54 (33.8-126)	0.720
Albumin (g/L)	31 (27-34)	28 (26.8-32)	0.338
Total bilirubin (µmol/L)	19 (14.8-32.8)	20 (11.2-24.8)	0.386
Creatinine (µmol/L)	91 (78-124.5)	77.5 (63.8-97)	0.052
Sodium (mmol/L)	137.5 (135-139)	136.5 (133.8-140)	0.733
Platelets (10^9/L)	218 (139-296.8)	239.5 (149.2-354)	0.624
GGT (IU/L)	197.5 (68.5-350)	126 (37.5-205)	0.052
Alk Phos (IU/L)	149 (84-194)	113 (80.5-213.5)	0.795
Hemoglobin (g/L)	130 (116.5-148.5)	113 (101.5-132.8)	0.015
INR	1 (1-1)	1 (1-1)	0.365
MELD	11 (9-14)	9 (6-12)	0.018
**Background liver**			
Histological F3-F4	20/36 (56%)	9/18 (50%)	0.777
Histological or clinical F3-F4	29/45 (64%)	12/22 (55%)	0.594
AFP (ng/mL)	10.5 (3.8-331.2)	8.5 (4-760.8)	0.775
Surveillance prior to diagnosis	4/36 (11%)	1/20 (5%)	0.645
**Cancer staging**			
T1	7/46 (15%)	7/24 (29%)	0.300
T2	20/46 (43%)	6/24 (25%)	
T3	17/46 (37%)	9/24 (38%)	
T4	2/46 (4%)	2/24 (8%)	
N0	29/33 (88%)	12/14 (86%)	1.000
N1	4/33 (12%)	2/14 (14%)	
N2	0/33 (0%)	0/14 (0%)	
M0	24/34 (71%)	12/16 (75%)	1.000
M1	10/34 (29%)	4/16 (25%)	
TNM stage			0.602
1 (T1N0M0)	5/34 (15%)	5/16 (31%)	
2 (T2N0M0)	10/34 (29%)	3/16 (19%)	
3 (T3-4N0M0)	9/34 (26%)	4/16 (25%)	
4 (N1 or M1)	10/34 (29%)	4/16 (25%)	
**Treatment**			
Liver transplantation	1/49 (2%)	1/24 (4%)	1.000
Resection	8/49 (16%)	7/24 (29%)	0.228

Radiofrequency ablation	5/49 (10%)	5/24 (21%)	0.281
Chemoembolization	23/49 (47%)	9/24 (38%)	0.466
Systemic therapy	6/49 (12%)	4/24 (7%)	0.720
Best supportive care / no therapy	14/49 (29%)	6/24 (25%)	1.000

Table S1. Comparison of clinical characteristics between female and male patients with NAFLD-HCC (n=76)

AFP, alpha fetoprotein; ALT, alanine transaminase; AST, aspartate aminotransferase; BMI, body mass index; GGT, gamma-glutamyl transferase; INR, international normalized ratio; LT, liver transplantation; MELD, Model for End Stage Liver Disease; RFA, radiofrequency ablation; TACE, transarterial chemoembolization

Variable	NAFLD (n=76)	MAFLD, no NAFLD (n=377)	no MAFLD, no NAFLD, (n=467)	Overall p-value
**Clinical**				
Age (y)	75 (70-80)	67 (60-74)	67 (58-77)	<0.001
Female sex (%)	24/76 (31.6%)	54/377 (14.3%)	103/467 (22.1%)	0.001
Diabetes (%)	54/74 (73%)	236/356 (66.3%)	1/242 (0.4%)	<0.001
Hypercholesterolemia (%)	34/72 (47.2%)	81/318 (25.5%)	19/233 (8.2%)	<0.001
Weight (kg)	81 (69.8-91)	80 (72.3-90.6)	66.7 (57-74)	<0.001
BMI (kg/m2)	28.7 (25.6-31)	27.4 (25.5-30)	21.4 (19.9-23.3)	<0.001
**Biological**				
ALT (IU/L)	36 (25-61)	48 (30-78.2)	51.5 (33-88)	0.007
AST (IU/L)	53 (37.5-95.2)	74 (48-124)	89 (60-151.5)	<0.001
Albumin (g/L)	30 (27-34)	29 (25-34)	28 (23-32)	0.011
Total bilirubin (µmol/L)	19 (14-31)	26 (17-46)	32 (18-62.8)	<0.001
Creatinine (µmol/L)	87.5 (73-117.8)	85 (70-112)	80 (65.5-100.5)	0.015
Sodium (mmol/L)	137 (134.8-139)	136 (134-139)	137 (134-139)	0.105
Platelets (10^9/L)	224.5 (142.2-305)	144 (99.2-199.2)	129 (83-208)	<0.001
GGT (IU/L)	167 (62-295)	203 (110.8-362.2)	182.5 (101-357.2)	0.171
Alk Phos (IU/L)	130.5 (82.8-196.8)	123 (90-186)	133 (97-197)	0.205
Hemoglobin (g/L)	126 (107-145.5)	128 (111.2-142)	127 (109-142)	0.753
MELD	10 (8-14)	12 (8-15.5)	12 (9-17)	0.075
Histological or clinical F3-F4	41/67 (61.2%)	294/312 (94.2%)	264/286 (92.3%)	<0.001
**Tumor characteristics**				
TNM stage 3 or 4	27/50 (54%)	130/205 (63.4%)	204/290 (70.3%)	0.046
AFP (ng/mL)	9.5 (4-463)	21 (6-315.5)	40 (7-745)	0.035
**First line therapy**				
Curative therapy (LT, Resection or RFA)	25/71 (35.2%)	91/340 (26.8%)	77/341 (22.6%)	0.070
Palliative therapy (TACE, systemic therapy or other)	26/71 (36.6%)	140/340 (41.1%)	109/341 (32.0%)	0.044
Best supportive care / no therapy	20/71 (28.2%)	109/340 (32.1%)	155/341 (45.4%)	<0.001

<u>Table S2.</u> Clinical characteristics of NAFLD subjects (all were also MAFLD), MAFLD without NAFLD and subjects without NAFLD or MAFLD

Variable	NAFLD (n=76)	HCV, MAFLD (n=82)	HCV, no MAFLD (n=109)	p-value	ALD, MAFLD (n=220)	ALD, no MAFLD (n=177)	p-value
**Clinical**							
Age (y)	75 (70-80)	62 (53-69)	59 (52-71)	0.871	68 (61.8-74)	67 (61-73)	0.380
Female sex (%)	24/76 (31.6%)	25/82 (30.5%)	35/109 (32.1%)	0.875	17/220 (7.7%)	18/177 (10.2%)	0.477
Diabetes (%)	54/74 (73%)	51/77 (66.2%)	0/71 (0%)	<0.001	141/212 (66.5%)	0/99 (0%)	<0.001
Hypercholesterolemia (%)	34/72 (47.2%)	17/72 (23.6%)	5/68 (7.4%)	0.010	55/193 (28.5%)	6/95 (6.3%)	<0.001
Weight (kg)	81 (69.8-91)	75.5 (66-85.8)	64.5 (55-71.2)	<0.001	82 (75-91.9)	68.2 (61-77)	<0.001
BMI (kg/m^2)	28.7 (25.6-31)	26.1 (24.8-28)	21.2 (19.9-23.2)	<0.001	28.4 (25.9-30.7)	22.4 (20.3-23.4)	<0.001
**Biological**							
ALT (IU/L)	36 (25-61)	66 (41-113)	69 (43-113)	0.773	40 (28-62.5)	41 (28-63.8)	0.938
AST (IU/L)	53 (37.5-95.2)	95 (59.8-126.5)	102 (72.5-158.5)	0.010	68 (45-119.2)	81 (55-129)	0.037
Albumin (g/L)	30 (27-34)	29 (24-33)	28 (24.8-32)	0.800	28.5 (25-34)	26.5 (23-30.8)	0.003
Total bilirubin (µmol/L)	19 (14-31)	24 (16-45)	24 (18-48)	0.439	27 (17-46.8)	41 (22-75)	0.002
Creatinine (µmol/L)	87.5 (73-117.8)	75.5 (66-104)	73 (61-88.5)	0.257	85 (72-110)	83 (67-114)	0.667
Sodium (mmol/L)	137 (134.8-139)	137 (134-139)	136 (135-139)	0.612	136 (133-139)	136 (133-139)	0.452
Platelets (10^9/L)	224.5 (142.2-305)	110.5 (79.2-173)	96 (69-137.8)	0.099	149 (111.8-197.8)	136.5 (87.8-205.2)	0.152
GGT (IU/L)	167 (62-295)	125.5 (74.2-253.2)	142 (75.2-242.5)	0.664	264 (134.8-390.2)	209 (146.5-428)	0.660
Alk Phos (IU/L)	130.5 (82.8-196.8)	116 (86.5-164)	125 (94.5-153)	0.461	127 (94.5-189.5)	143 (105-212)	0.038
Hemoglobin (g/L)	126 (107-145.5)	125 (113-139)	128 (114.8-143.2)	0.186	128 (109-142)	123 (104-138)	0.059
MELD	10 (8-14)	12 (8-15)	10 (8-16)	0.787	12 (8-16)	13 (10-18)	0.020
Histological or clinical F3-F4	41/67 (61.2%)	72/75 (96%)	91/92 (98.9%)	0.327	174/185 (94.1%)	118/127 (92.9%)	0.815
**Tumor characteristics**							
TNM stage 3 or 4	27/50 (54%)	23/48 (47.9%)	42/75 (56%)	0.460	76/113 (67.3%)	75/107 (70.1%)	0.666
AFP (ng/mL)	9.5 (4-463)	24.5 (8-225.5)	42 (10-413.5)	0.436	15 (5-318)	28 (6-824)	0.152
**First line HCC therapy**							

Curative therapy (LT, Resection or RFA)	25/71 (35.2%)	31/78 (39.7%)	30/100 (30.0%)	0.204	43/202 (21.3%)	22/141 (15.6%)	0.209
Palliative therapy (TACE, systemic therapy or other)	26/71 (36.6%)	34/78 (43.6%)	40/100 (40.0%)	0.649	84/202 (41.6%)	43/141 (30.5%)	0.041
Best supportive care / no therapy	20/71 (28.2%)	13/78 (16.7%)	30/100 (30%)	0.052	75/202 (37.1%)	76/141 (53.9%)	0.003

Table S3. Comparison of HCV and ALD subjects with and without MAFLD. P-values refer to comparisons between MAFLD vs no MAFLD across aetiology.

Variable	NAFLD-HCC (definition 1), n=76	NAFLD (definition 2), n=101
**Clinical**		
Age (y)	75 (70-80)	76 (71-82)
Female sex (%)	24/76 (31.6%)	32/101 (31.7%)
Diabetes (%)	54/74 (73%)	54/95 (56.8%)
Hypercholesterolemia (%)	34/72 (47.2%)	36/93 (38.7%)
Weight (kg)	81 (69.8-91)	76 (66.7-90)
BMI (kg/m2)	28.7 (25.6-31)	28.5 (24.9-30.8)
BMI ≥ 25kg/m2 (%)	48/73 (65.8%)	51/95 (53.7%)
**Biological**		
ALT (IU/L)	36 (25-61)	36 (24.8-59.2)
AST (IU/L)	53 (37.5-95.2)	54 (36-101.5)
Albumin (g/L)	30 (27-34)	29 (27-34)
Total bilirubin (μmol/L)	19 (14-31)	20 (14-32)
Creatinine (μmol/L)	87.5 (73-117.8)	86 (73-116.2)
Sodium (mmol/L)	137 (134.8-139)	137.5 (135-140)
Platelets (109/L)	224.5 (142.2-305)	225 (141-294)
GGT (IU/L)	167 (62-295)	167 (64-342)
Alk Phos (IU/L)	130.5 (82.8-196.8)	147 (82.2-208)
Hemoglobin (g/L)	126 (107-145.5)	126.5 (107.8-141.8)
INR	1.19 (1.03-1.64)	1.20 (1.00-1.50)
MELD	10 (8-14)	10 (8-14)
**Background liver**		
Histological F3-F4	29/54 (53.7%)	30/60 (50%)
Histological or clinical F3-F4	41/67 (61.2%)	54/85 (64%)
AFP (ng/mL)	9.5 (4-463)	12 (4-510.2)
Surveillance prior to diagnosis	5/56 (9%)	5/63 (8%)
**Median survival** (weeks)	101 (46-160)	72 (39-122)
**Cancer TNM** stage		
1 (T1N0M0)	10/50 (20%)	12/61 (20%)
2 (T2N0M0)	13/50 (26%)	16/61 (26%)
3 (T3-4N0M0)	13/50 (26%)	15/61 (25%)
4 (N1 or M1)	14/50 (28%)	18/61 (30%)
**Therapy**		
Curative therapy (LT, Resection or RFA)	25/73 (34%)	26/93 (28%)
Palliative therapy (TACE, systemic therapy or other)	44/73 (60%)	49/93 (53%)
Best supportive care / no therapy	20/73 (27%)	34/93 (37%)

Table S4. Comparison of different definitions of NAFLD-HCC. *Definition 1* corresponds to exclusion of other causes of liver disease and histological / clinical documentation of NAFLD or presence of metabolic syndrome / diabetes or obesity. *Definition 2* additionally includes patients with cryptogenic liver disease (see Methods for details).

Supplementary Figures

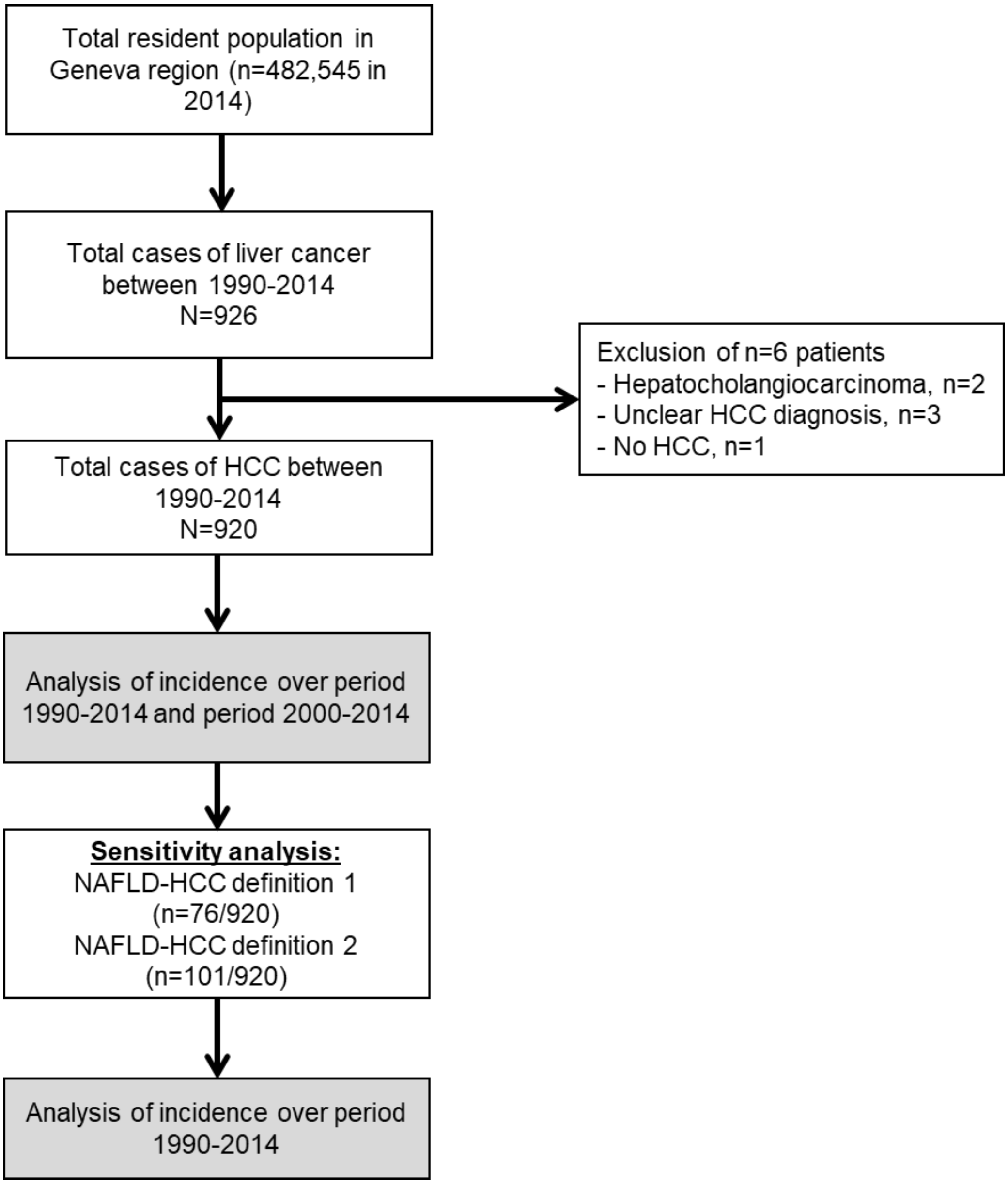

Fig. S1. Flowchart of the study population.

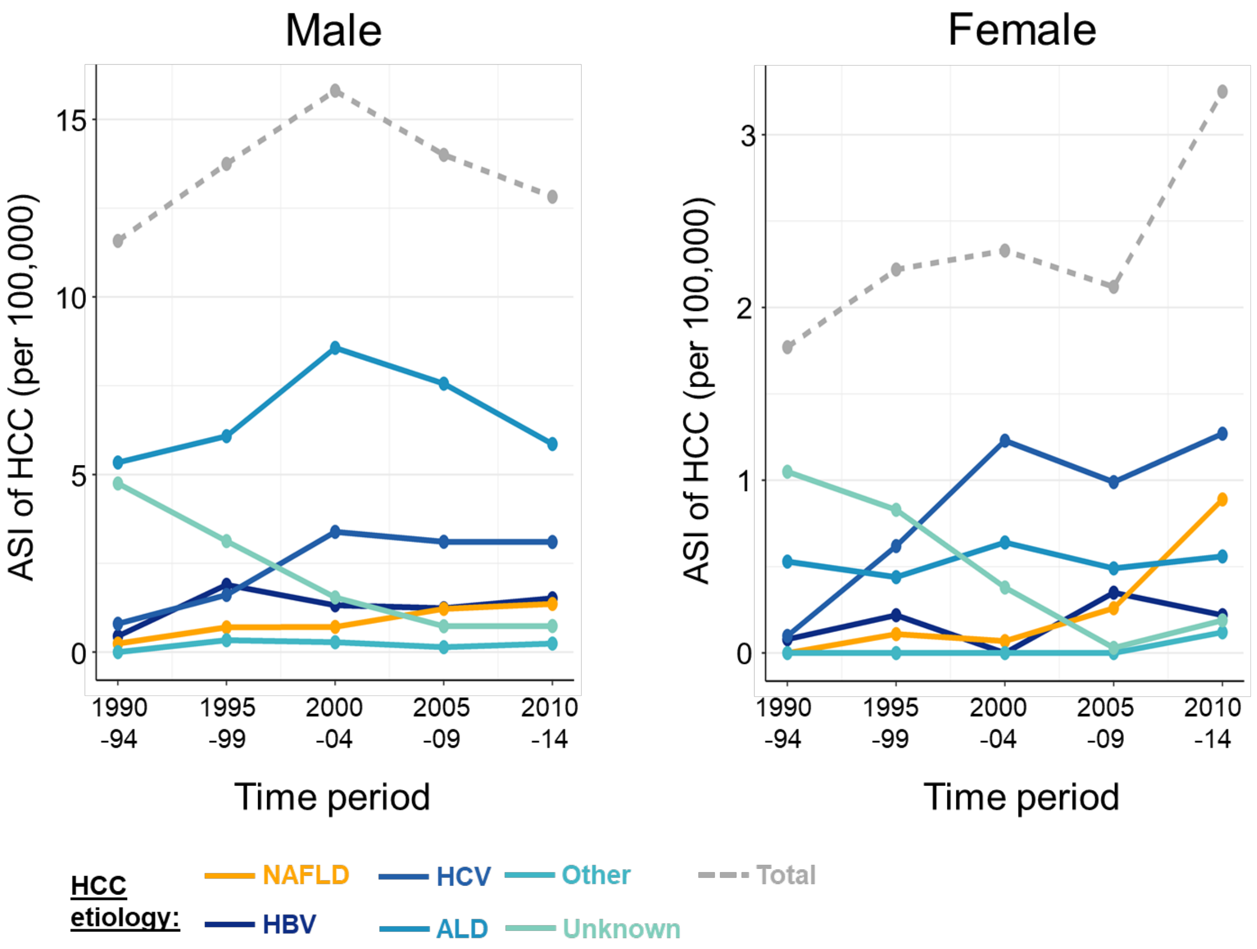

Fig. S2. Age-standardised incidence of HCC by etiology and according to male and female sex in the canton of Geneva in 5-year periods from 1990 to 2014.

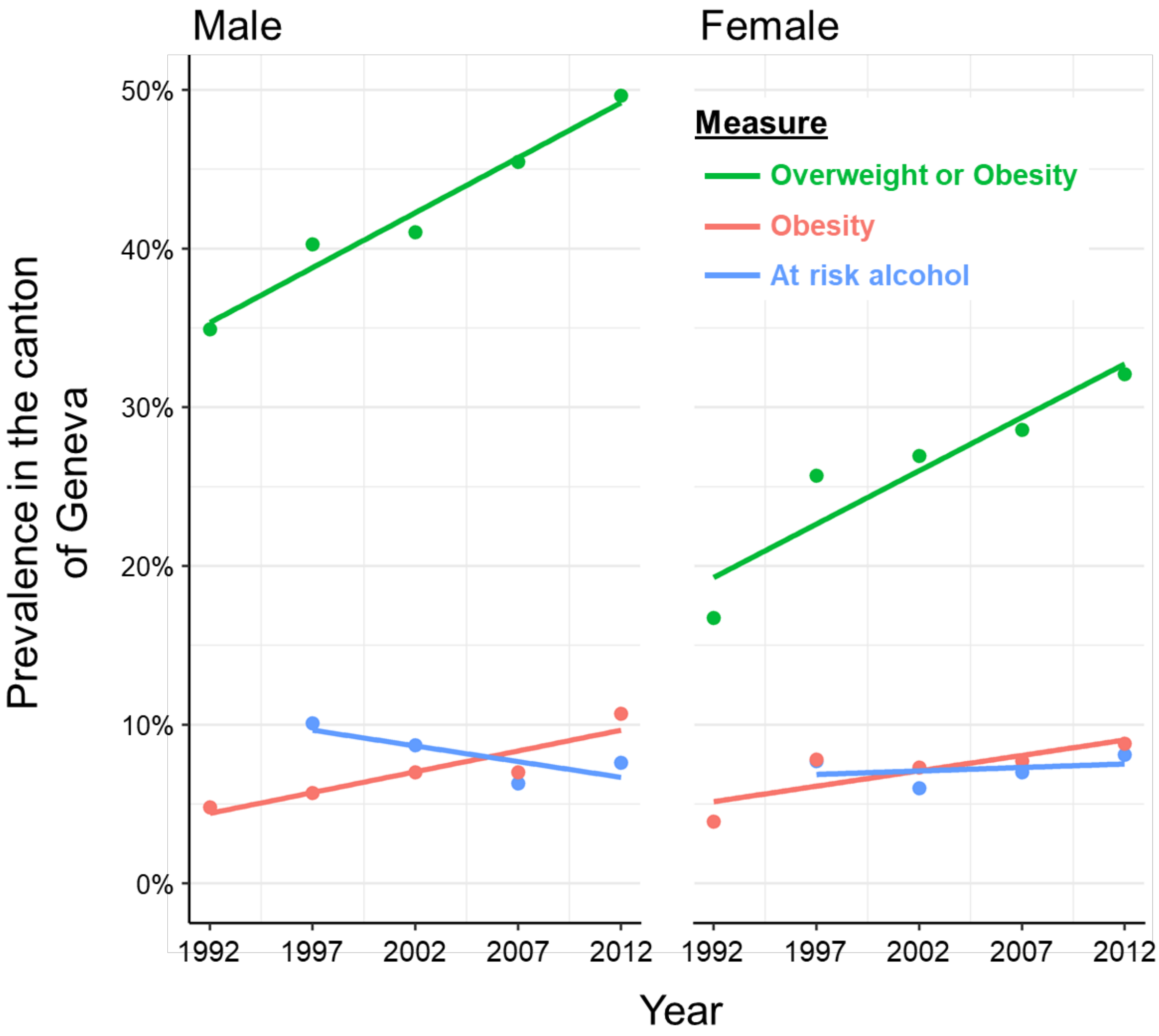

Fig. S3. Prevalence of overweight or obesity (body mass index [BMI] ≥ 25 kg/m^2), obesity (BMI] ≥ 30 kg/m^2) and at risk alcohol consumption in the canton of Geneva from 1992 to 2012 in men and women. At risk alcohol consumption is defined as 4 standard units or more for men and 2 standard units or more for women a day.

Data from: Enquête suisse sur la santé 2017. Office fédéral de la statistique; 2018. https://www.ge.ch/statistique/ofs/sante2017/files/fr/02.xml accessed April 10th 2020.

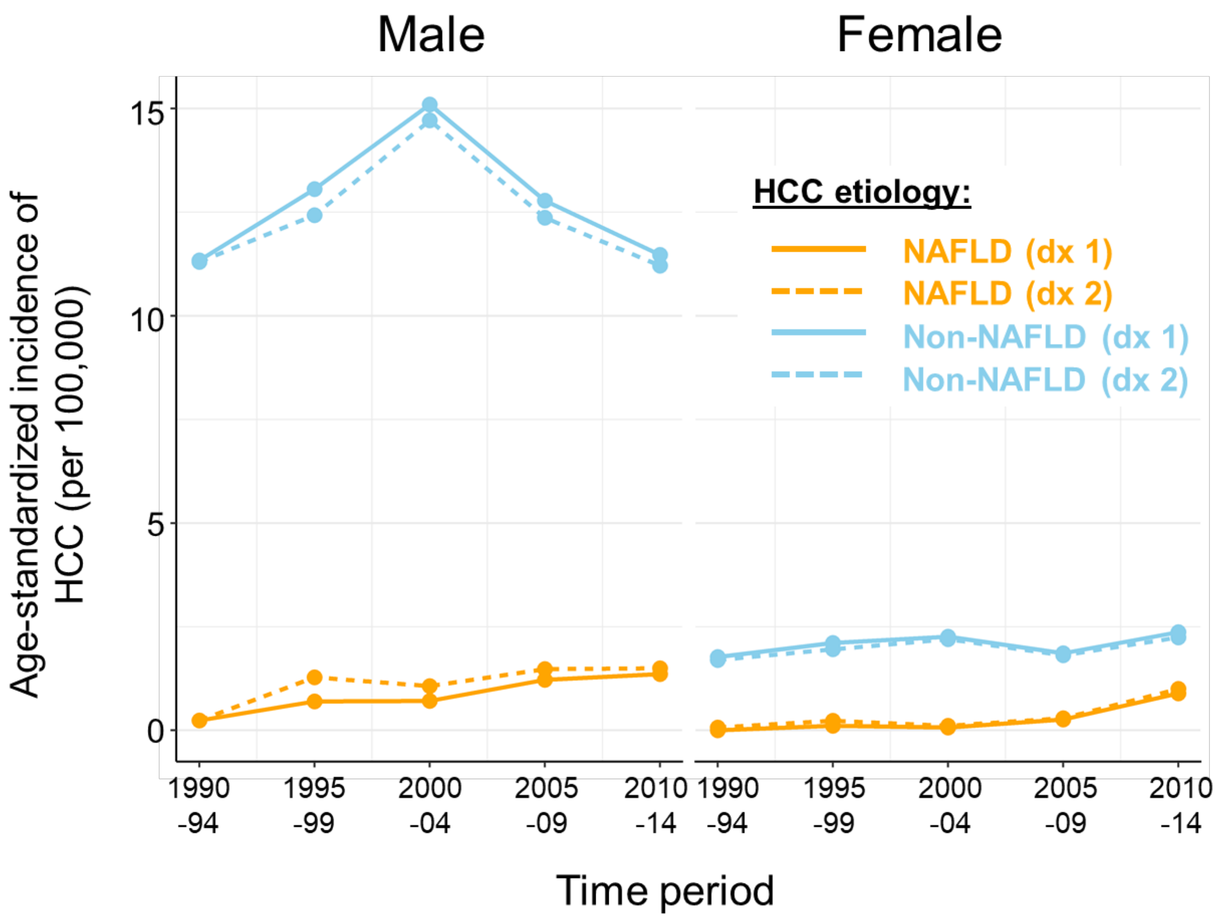

Fig. S4. Age-standardised incidence of HCC according to NAFLD-HCC diagnostic criteria. Definition 1 (dx 1) corresponds to exclusion of other causes of liver disease and histological / clinical documentation of NAFLD or presence of metabolic syndrome / diabetes or obesity. Definition 2 (dx 2) additionally includes patients with cryptogenic liver disease (see Methods for details).
