# Supplementary material for: NAFLD and MAFLD as emerging causes of HCC: A populational study
Source: JHEP Rep. 2021 Jan 19;3(2):100231. doi: 10.1016/j.jhepr.2021.100231 (PMC7957147; doi:10.1016/j.jhepr.2021.100231)
Supplement: JHep_Reports_CTAT_table.pdf [file mmc2.pdf]

JHEP|Reports

JHEP Reports

CTAT methods

Tables for a "Complete, Transparent, Accurate and Timely account" (CTAT) are now mandatory for all revised submissions. The aim is to enhance the reproducibility of methods.

- Only include the parts relevant to your study
- Refer to the CTAT in the main text as 'Supplementary CTAT Table'
- Do not add subheadings
- Add as many rows as needed to include all information
- Only include one item per row

If the CTAT form is not relevant to your study, please outline the reasons why:

1.1 Antibodies

Name	Citation	Supplier	Cat no.	Clone no.

1.2 Cell lines

Name	Citation	Supplier	Cat no.	Passage no.	Authentication test method

1.3 Organisms

Name	Citation	Supplier	Strain	Sex	Age	Overall n number

1.4 Sequence based reagents

Name	Sequence	Supplier

1.5 Biological samples

Description	Source	Identifier

1.6 Deposited data

Name of repository	Identifier	Link

1.7 Software

Software name	Manufacturer	Version
**R**	R core team	**3.4.4**

1.8 Other (e.g. drugs, proteins, vectors etc.)

1.9 Please provide the details of the corresponding methods author for the manuscript:

Dr Nicolas Goossens, MD, MSc, PD
Division of Gastroenterology and Hepatology,
Department of Medicine
Geneva University Hospital
Rue Gabrielle-Perret-Gentil 4, 1205 Geneva
Geneva, Switzerland
Telephone: +41.22.3729350, Fax: +41.22.3729021
Email: nicolas.goossens@hcuge.ch

2.0 Please confirm for randomised controlled trials all versions of the clinical protocol are included in the submission. These will be published online as supplementary information.
